# The economic impact of compassionate use of medicines

**DOI:** 10.1186/s12913-021-07255-w

**Published:** 2021-12-04

**Authors:** Claudio Jommi, Federico Pantellini, Lisa Stagi, Maria Verykiou, Marianna Cavazza

**Affiliations:** 1 Centre for Research on Health and Social Care Management, SDA Bocconi School of Management, CERGAS, Bocconi University, Via Sarfatti 25, 20136 Milano, MI Italy; 2 ROCHE Spa, Viale GB Stucchi 110, MB 20900 Monza, Italy

**Keywords:** Compassionate Use, Medicines, Economic Impact, Italy

## Abstract

**Background:**

Compassionate use programs (CUP) for medicines respond to the ethical imperative of providing access to medicines before marketing approval to patients not recruited in trials. The economic impact of clinical trials has previously been investigated. No evidence on the net economic benefit of CUP exists. This research aims to address this information gap by estimating the economic consequences of 11 CUP in Italy conducted between March 2015 and December 2020 from the perspective of public health care system in Italy (National Health Service). Eight programs concern cancer treatments, two refer to spinal muscular atrophy, and one is indicated for multiple sclerosis.

**Methods:**

Since CUP medicines are covered by the industry, the net economic benefit includes: (i) avoided costs of the Standard of Care (SoC) the patients would have received had they not joined the CUP, (ii) costs not covered by the pharmaceutical industry sponsor, but instead sustained by payers, such as those associated with adverse events (only severe side effects resulting in hospitalisation and attributable to CUP medicines), and (iii) costs for combination therapies and diagnostic procedures not used with the SoC. The SoC costing relied on publicly available data. Information on adverse events and diagnostic procedures was retrieved from the CUP and monetized using the relevant fee for episode or service. One CUP was excluded since a SoC was not identified.

**Results:**

2,713 patients were treated in the 11 CUP where a SoC was identified. The SoC mean cost per patient ranged from €11,415 to €20,299. The total cost of the SoC ranged between €31.0 and €55.1 million. The mean cost per patient covered by hospitals hosting CUP was equal to €1,646, with a total cost of €4.5 million. The net economic benefit ranged €26.5 million - €50.6 million.

**Conclusions:**

Despite research limitations, this paper illustrates for the first time the net economic impact of CUP from a public payer perspective. It is important to integrate these estimates with the prospective effects of CUP implementation, i.e., the economic value of the comparative benefit profile of medicines used in CUP versus the SoC, including effects from a societal perspective.

**Supplementary Information:**

The online version contains supplementary material available at 10.1186/s12913-021-07255-w.

## Background

Access to unauthorized treatments can be achieved through participation in clinical trials (CT) or through early access programs. The latter includes different mechanisms, and different terminologies often to the same effect, such as individual named-patient, managed access, compassionate use, early, pre-approval, or expanded access programs [1].

Compassionate Use Programs (CUP) allow for the unauthorized use of a medicine outside a CT, where the cost of treatment is borne by the pharmaceutical sponsor. While the primary objective of a CT is to generate evidence on the safety and efficacy of a treatment against an unmet need, that of a CUP is addressing an unmet medical need for ethical reasons [1]. According to the Regulation (EC) No 726/2004 of the European Parliament, a CUP allows “a group of patients with a chronically or seriously debilitating disease or whose disease is considered to be life-threatening, and who cannot be treated satisfactorily by an authorised medicinal product” to have access to a product that is the object of an application for marketing authorisation or of a CT [2, 3].

Until recently, patients in Italy with a specific disease with no treatment option could be provided access, through their physicians, to innovative drugs authorised for use in any other country, unauthorised drugs under CT, as well as drugs different from those authorised for the intended therapeutic indication (off-label) [4]. In 2017, a new decree from the Italian Ministry of Health came into effect [5] reflecting the above EU regulations, while also authorising, as previously, the use in CUP of off-label licensed products and EC-approved products not yet licensed in Italy. By way of this decree, the definition encompasses access to unauthorized medicines, on a named (individual) basis as well as in a group program through their physicians, and to patients who suffer from severe and rare diseases or life-threatening conditions. Patients should have no other valid therapeutic alternatives, or cannot be recruited in a CT or had previously participated in a CT and demonstrated positive health outcomes, and can in this way be guaranteed continuity of care outside the CT. Authorization for conducting a CUP is based on evidence from ongoing Phase III CT, Phase II for life threatening or severe diseases, and Phase I for drugs for rare diseases or rare cancers, provided that Phase I results demonstrate efficacy and safety of the medicinal product at a given dosage and schedule of administration. CUP covers drugs before marketing authorisation, but in exceptional circumstances it can be prolonged as long as they are reimbursed by the National Health Service.

The use of unapproved drugs outside of clinical trials through compassionate use is the subject of debate among scientific community from different perspectives. Through CUP, pharmaceutical companies can respond rapidly and efficiently to the demand for an unmet need prior to authorization, thus streamlining the transition and launch of the product to market with real-world data [4]. These data may play an important role in regulatory decisions and can be incorporated in the registration and pricing of a product [4]. Provided that the treatment works, patients' health is benefited, on the other hand, by accessing a treatment earlier in the evolution of their pathology, and thus avoiding health deterioration due to long waiting times until official market authorization [6]. Evidence from the Netherlands suggests that patients who suffer from chronic or life-threatening diseases actively seek alternative options through CUP despite their varying level of understanding and information regarding availability and access [7]. On the other hand, doctors in the Netherlands reportedly have a more critical perception of CUP, in the form of expanded access program. They quote practical hurdles related to the application process and the moral issue of providing an early access to medicines that may not be approved or approved for the indication different from the one of CUP [8]. The point has been raised that with increasing awareness of the efficacy of new drugs through internet platforms and with potential inequities in pre-approval treatment access, healthcare providers, policy makers and pharmaceutical industry should find common solutions to manage patients' expectations in a fair and ethical way [9]. Finally, CUP can bring added value to society and third-party payers in economic terms, who may benefit from avoiding part of the cost of treating a severely ill patient with the existing standard of care (SoC) -whose relative effectiveness is often questionable- by instead shifting the burden to industry, which will cover most of the investigative treatment’s costs under the CUP.

Our research looked at CUP from the economic view and the perspective of the Italian public health care system. This system was shaped, since 1978, as a National Health Service (NHS) model, where the State is the most important financer, via general tax levies. A high proportion of health care expenditure is covered by the NHS (76.1 % in 2019) [10]. The pharmaceutical market is highly regulated, with price and reimbursement simultaneously negotiated by the National Medicines Agency and the pharmaceutical companies [11]. Medicines used in hospital settings are fully reimbursed by the NHS, whereas retail drugs are subject to co-payment and non-prescription medicines are not reimbursed. The NHS reimbursed 71 % of total pharmaceutical expenditure in 2020 [12].

More specifically, our research questions are: what costs would have been incurred by payers for treating patients had the latter not been part of a CUP (averted costs thanks to CUP)? What are the incremental costs incurred by the National Health Service due to patients’ inclusion in a CUP (incremental costs due to CUP)?

## Methods

We firstly carried out a literature review of the evidence on the economic impact of CUP. More specifically, a non-exhaustive review was performed on PubMed and Google Scholar to identify relevant research presenting evidence on third-party payer avoided and sustained costs for treatment of patients enrolled in CUP. Keywords used in the search included: “cost avoidance” (or) “economic impact” (or) “cost saving” (or) “economic value” (and) “compassionate use” (or) “early access”. As no specific papers were found on CUP, we extended the analysis to “clinical trial(s)”, to scrutinise methods and findings of these studies.

The empirical analysis was carried out on a database on CUP launched by Roche in Italy from 2015 to 2020. The CUP database includes anonymised information on patients (gender and age), compassionate use treatments (CUT), medicines used in combination (duration, dose), side effects classified according to MedDRA® [13], their severity, and whether, according to the company providing the CUT and/or by the hosting healthcare centre, they were associated with the CUT. We have considered only severe adverse events associated with the CUT in the case where they resulted in hospitalization.

For the purposes of this study, averted costs are defined as the SoC costs that would have otherwise been covered by healthcare providers if patients had not been included in the CUP. Averted costs of the SoC were estimated in three steps:

- we identified programs where the SoC did not exist when the patient started the CUT and eliminated from the analysis;
- the SoC for all other CUT was identified on the grounds of pivotal studies for CUT (where SoC was used as an active comparator), European/National Guidelines, or Regional Documents, and was validated by clinicians;
- the mean SoC cost per patient was estimated, i.e., unit price per dose * mean number of doses. In Italy, as in many other countries, public prices do not necessarily coincide with actual prices paid by hospitals, due to hidden discounts negotiated at central and regional levels and/or the effects of financial or outcome-based managed entry agreements (MEA) [11]. In 2017, discounts and MEA accounted for 25 % and 12 % of public price, respectively, for all medicines procured by hospitals [14]. As this information is not publicly available for each single medicine, we relied on actual prices published in regional documents, where those were available. Where they were not, we calculated the cost as the public price due for all non-innovative medicines, i.e., net of the compulsory discounts (5 %+5 %) per mg * unit dose retrieved from Summary of Product Characteristics (SmPC) in mg * median/mean treatment duration derived from SmPC or pivotal trials or other documents.

Incremental costs incurred by the CUP include:

- costs of medicines given in combination with CUT not covered by the company providing CUT for CUP;
- costs of diagnostic procedures incurred due to CUT but not covered by the company (procedure outside of normal clinical practice) which would not have been otherwise incurred with the SoC;
- costs of severe (i.e. generating hospitalisation) side effects associated with the CUT by the company providing the CUT and/or by the hosting healthcare centre.

The cost of combination therapy not covered by the company providing the CUT was calculated as the price for each patient, net of compulsory discounts (5+5 %), * unit dose from the CUP dataset * treatment duration from the CUP dataset.

In Italy, diagnostic procedures and inpatient care are reimbursed through fee-for-service and fee-for-episode schemes, respectively [15]. The fee-for-service for ambulatory care was monetized using the most updated regional fees (Lombardy and Veneto) [16, 17], because national fees have not been updated since 1996 [18]. Hospitalisation fees were retrieved from the national database and are up to date as of 2012. Hospitalisations are classified according to the DRG – Diagnostic-Related Group – system (ICD-9-CM 2007 version and Medicare DRG classification 24th version) [19]. DRG classification of inpatient episodes does not correspond with the one used by MedDRA®. We relied on the e-DRGs platform [20] to associate the MedDRA® denomination with the relevant DRG (and the corresponding fee).

## Results

### Literature review on economic impact of clinical trials

A total of 21 studies were identified, with most evidence published for Spain and the USA (United States of America) (4 papers each), followed by Canada and Italy (3 papers each), while single publications included data for Australia, Austria, France, Germany, Taiwan, Turkey, and UK (United Kingdom).

Of these studies, one pertained to the measurement of costs of specific side-effects during CT [41], one comprised a grey literature report on the total impact and savings incurred by CT in the USA [40], and one reported on additional costs incurred by CT versus SoC [28]. These studies were excluded from the following description of available evidence of CT avoided costs but are included as references in Table 1.

**Table 1:** Evidence on the economic impact of Clinical Trials

Ref. ID	Country	Therapeutic Area	# of trials	Phase	# of patients	Year(s)	Cost items included (medicines, diagnostics, side effects, trial management, others)	Methods (clinical trial data / clinical trial protocol / mixed)	Main findings (total cost avoided / mean cost avoided per patient recruited)
[24]	Australia	Haematology	36	I, II, III	245	2006-2017	Medicines	Clinical trial data	€4,278,116 total cost avoided
[25]	Austria	Multiple	1,029	I, II, III, IV	23,331	2012-2017	Medicines, diagnostics, side effects, others	Clinical trial protocol	€100.5mi saved annually
[26]	Canada	Cancer	21	III	4,674	1999-2011	Medicines, diagnostics	Clinical trial protocol	The total Drug Cost Avoidance (DCA) was estimated at €20,308,422 of which targeted therapy constituted 43 % (five trials). The combined Pathology Cost Avoidance (PCA) and DCA was €23,356,118 for a cost avoidance per patient of €5,447.83
[27]	Canada	Cancer	37	I/II, II, III	250	2001-2006	Medicines	Clinical trial protocol	Drug specific cost avoidance per patient: €7.99 - €169,885.51
									Potential drug specific cost avoidance per patient: €9.62 - €195,000.48
									Actual drug cost avoidances according to tumour group were calculated showing a median range of €936.74 - €16,157.14 per patient between tumour groups. The median range for potential drug cost avoidance was substantially higher from €6,712.93 - €31,727.89 per patient
[28]^**0**^	Canada	Breast cancer	8	III	97	2006-2009	Medicines, diagnostics, trial management, others	Clinical trial data	Mean additional total costs between CT and SoC patients of €4,601 (95 % confidence interval: €94 - €9,109 *p*=0.046)
[29]	France	Oncology - Haematology	27	III	177	2011-2016	Medicines	Mixed	Total cost savings were €5.2mi
									Mean cost saving per patient was €19,182.7 ± €29,865.7
[23]	Germany	Oncology	88	Un-specified	Un-specified	2002-2005	Medicines	Mixed	€5.1mi potential drug cost savings
									€1.5mi actual drug cost savings
[30]	Italy	Lung cancer	12	Un-specified	44	2010	Medicines, diagnostics	Clinical trial contract	€243,154 drug cost savings
[31]	Italy	Oncology	34	I, II, III	126	2017	Medicines	Clinical trial protocol	Average hospital saving of €5,487 per patient treated in pharma sponsored studies and €206 for investigator-led studies
									€517,658 in a month for drugs that otherwise would have been loaded on the Italian National Health Service
[32]	Italy	Oncology - Haematology	29	II, III	189	2011-2016	Medicines	Mixed	Total avoided costs of €330,000
									Potential total avoided costs at national level would range from 320 to 360 million €/year
[33]	Spain	Lung cancer	12	I, II, III	69	2016	Medicines	Clinical trial data	The overall avoided cost was €474,428.65. The average cost per clinical trial was €39,535.72 and per patient was €6,875.77
[34]	Spain	Prostate cancer	5	III	136	1996-2013	Medicines	Clinical trial data	€696,002 total cost avoidance
									€139,200 average cost avoidance per clinical trial
									€5,118 average cost avoidance per patient
[35]	Spain	Breast cancer	37	I, II, III	89	2014-2016	Medicines	Clinical trial protocol	80 % of cost savings were derived from phase III trials
									€957,246 total cost avoidance
									€10,756 average cost avoidance per patient
[36]	Spain	Oncology	38	Un-specified	261	2017-2018	Medicines	Clinical trial (unspecified)	Avoided cost: €3,482,662 / year; €13,343/patient
[37]	Taiwan	Multiple	194	I, II, III, IV	2,883	2008	Medicines	Clinical trial data	Average cost avoidance of €39,456/trial-year or €26,531/participant-year
[38]	Turkey	Multiple	174	I, II, III, IV	1,437	2006-2010	Medicines	Clinical trial data	€212,478,657 government saving
[39]	UK	Oncology	53	II, III	357	2009-2010	Medicines, diagnostics, trial management, others	Clinical trial protocol	€436,763 (2009) and €344,833 (2010) overall treatment cost savings
[40]*	USA	Multiple	6,199	0, I, II, III, IV	1,100,000	2013	Medicines, diagnostics, trial management, others	Clinical trial data	Estimates of Overall Economic Impact of Industry-Sponsored Clinical Trial Activities at U.S. Trial Sites 2013
									Direct – Research activities at clinical trial sites around the country €7.4 bn
									Indirect and Induced – Vendors and suppliers to trial sites; Consumer purchases by researchers and workers engaged in or supporting the clinical trial process €11.4 bn
									Total €18.8 bn
[41]^**§**^	USA	Lung cancer	4	III	31	2017	Medicines, diagnostics, trial management, side effects, others	Clinical trial data	The mean cost to treat an event of grade 3 nausea was €12,135
									The mean costs to treat an event of grade 3–4 thrombocytopenia combined excluding and including hospitalization costs were €544,885 (SD = €1,283.15) and €3,678 (SD = €8,418.80) respectively
[22]	USA	Oncology and AIDS	255	Un-specified	756	1996-97	Medicines	Clinical trial data	€2.7 mi cost avoidance in drug costs
[21]	USA	Multiple	107	Un-specified	Un-specified	2000-2002	Medicines	Mixed	Mean drug cost avoidance €2,417,117 per year

^*0*^*Additional costs incurred by CT versus SoC reported*

*Grey literature report on cumulative economic impact of industry trials in the USA in one year

^*§*^*Side-effects costs*

In the remaining 18 publications reporting CT avoided costs, the number of CT included in each paper ranged from 4 to 1,029, with recruitment of a total of 44 to 23,331 patients. The majority (78 %) of the publications report savings on medicine costs, while the rest also reported on diagnostics, clinical trial management, side effects, and other cost items. Costs were converted from the reported currencies to Euros using the average exchange rate of the year of the most recent data presented in each paper. Out of the 18 studies, six used clinical trial data, six extracted data from protocols, four studies employed mixed methods (data and protocols), one extracted data from clinical trial contracts, while for one study the source of data was not available. The average cost savings on all cost items reported in the available evidence range from €1,095 to €49,301 per patient and from €7,374 to €968,000 per clinical trial. Avoided drug costs reported in studies measuring only medicine costs ranged from €1,746 to €49,301 per patient and from €10,588 to €193,259 per clinical trial. The total reported cost savings ranged from €4,601 (97 patients in CT) to €212.5 million (14,370 patients in CT). Finally, three studies [25, 32, 38] were able to provide projected annual cost avoidance estimates on a national scale, which averaged to €217.7 million per year. As evidenced in Table 1, studies were included that reported total costs for multiple pathologies and multiple clinical trial phases. This is one of the reasons for the large range of cost avoidance found in the literature, combined with other factors such as the duration patients remain on a trial, which may vary across therapeutic areas, the number of days in each cycle a drug is administered, the number of clinic visits, and the price of the drugs.

### Empirical analysis

The Database on CUP launched by Roche in Italy from 2015 to 2020 includes 11 program and 2,745 patients (53 CUP were available in Italy in October 2021) [68]. These programs concerned 8 medicines / indications for cancer (1,641 patients, 59.8 % of all patients), one for primary progressive multiple sclerosis (1,045, 38.1 % of patients), and two for spinal muscular atrophy (59, 2.1 % of all patients) (Table 2).

**Table 2:** Compassionate Use Programs available in the CUP Database

# of CUP	Molecule	Indication	Indication (short)	# of patients	Starting month of CUP
MO29499	Alectinib	Adult patients with Anaplastic Lymphoma Kinase (ALK)-positive advanced Non-Small Cell Lung Cancer (NSCLC)	Non-Small Cell Lung Cancer 1	21°	March 2015
ML40066	Alectinib	Adult patients with ALK-positive advanced NSCLC, previously treated with crizotinib	Non-Small Cell Lung Cancer 2	226°	May 2017
ML39740	Atezolizumab	Adult patients with locally advanced or metastatic Urothelial Carcinoma (UC) after prior platinum-containing chemotherapy, or considered cisplatin ineligible, and whose tumours have a PD-L1 expression ≥ 5 %	Urothelial Carcinoma	222°	February 2017
AL41528	Atezolizumab	Adult patients with locally advanced or metastatic NSCLC after prior chemotherapy (patients with EGFR mutant or ALK-positive NSCLC should also have received targeted therapies before)	Non-Small Cell Lung Cancer	125°	June 2019
AL41712	Atezolizumab	Adult patients with unresectable locally advanced or metastatic Triple-Negative Breast Cancer (TNBC), whose tumours have PD-L1 expression ≥ 1 % and who have not received prior chemotherapy for metastatic disease	Triple-Negative Breast Cancer	41°	November 2019
M029476	Cobimetinib	Adult patients with unresectable or metastatic Melanoma with a BRAF V600 mutation	Melanoma	228°	May 2015
MA30130	Ocrelizumab	Adult patients with Primary Progressive Multiple Sclerosis (PPMS) (in terms of disease duration and level of disability) and with imaging features characteristic of inflammatory activity	Primary Progressive Multiple Sclerosis	1,045°	June 2017
AG40852	Entrectinib	Adult and paediatric patients 12 years of age and older with solid tumours expressing a Neurotrophic Tyrosine Receptor Kinase (NTRK) gene fusion, who have a disease that is locally advanced, metastatic or where surgical resection is likely to result in severe morbidity, and who have not received a prior NTRK inhibitor or who have no satisfactory treatment options	Solid tumours (NTRK)	1	August 2019
		Adult patients with ROS1-positive advanced NSCLC, not previously treated with ROS1 inhibitors	Non-Small Cell Lung Cancer	5°	
AG40661	Polatuzumab Vedotin	Adult patients with relapsed/refractory Diffuse Large B-Cell Lymphoma (DLBCL) who are not candidates for haematopoietic stem cell transplant.It is indicated in combination with the treatment of adult patients with bendamustine and rituximab	Diffuse Large B-Cell Lymphoma	151°	May 2019
AG41381	Risdiplam	Patients from 2 months old with 5q spinal muscular atrophy (SMA) Type 1, Type 2 or Type 3, or those who have up to 4 copies of SMN2 gene (Type 1)	Spinal Muscular Atrophy Type 1	28°	January 2020
AG42025	Risdiplam	Patients from 2 months old with 5q Spinal Muscular Atrophy (SMA) Type 1, Type 2 or Type 3, or those who have up to 4 copies of SMN2 gene (Type 2)	Spinal Muscular Atrophy Type 2	31	January 2020
AL41711	Trastuzumab emtansine	Adult patients with HER2-positive, unresectable locally advanced or metastatic Breast Cancer who previously received trastuzumab and a taxane, separately or in combination. Patients should have either: (i) received prior therapy for locally advanced or metastatic disease, or (ii) developed disease recurrence during or within six months of completing adjuvant therapy.	Breast Cancer	621°	September 2019
**Total # of patients**				**2,745** **° 2,713 included in the analysis**	

Two programs were excluded from the analysis since the SoC was not available: risdiplam for Type 2 Spinal Muscular Atrophy – SMA (31 patients) and entrectinib for solid tumours expressing a neurotrophic tyrosine receptor kinase – NTRK - gene fusion (1 patient). Data were elaborated for 2,713 patients.

Input data are illustrated in Table 3: identified SoC; combination therapies - whether they are covered by the company providing the CUT or the NHS; diagnostic tests - whether they are covered by the company providing the CUT or used also with the SoC or covered by the NHS; and hospitalisations due to side effects of the CUT or the relevant combination therapy. Side effects of CUT or combination therapies resulted in hospitalisation for 2.1 % of patients.

**Table 3:** Input data for the economic impact evaluation of CUP

# of CUP	Molecule	Indication (short)	Standard of Care	Combination medicines		Diagnostic test	Hospitilisations for side effects*	
MO29499	Alectinib	Non-Small Cell Lung Cancer 1	Ceritinib (1) Crizotinib (2)	-		Partially used with the SoC	0/21	
MO40066	Alectinib	Non-Small Cell Lung Cancer 2	Docetaxel (1) Pemetrexed low dosage (2) Pemetrexed high dosage (3) Ceritinib (4)	-		Used with the SoC	0/226	
ML39740	Atezolizumab	Urothelial Carcinoma	Docetaxel (1) Nivolumab (2) Pembrolizumab (3)	-		Used with the SoC	7/222 (3.2 %)	
AL41528	Atezolizumab	Non-Small Cell Lung Cancer	Pemetrexed low dosage (1) Pemetrexed high dosage (2) Bevacizumab+carboplatino+paclitaxel (3)	-		Used with the SoC	1/125 (0.8 %)	
AL41712	Atezolizumab	Triple-Negative Breast Cancer	Nab-paclitaxel	Nab-paclitaxel	Covered by the sponsor	Covered by the sponsor	2/41 (4.9 %)	
M029476	Cobimetinib	Melanoma	Nivolumab (1) Vemurafenib (2) Dabrafenib + trabetinib (3)	Vemurafenib	Not covered by the sponsor	Used with the SoC	14/228 (6.1 %)	
MA30130	Ocrelizumab	Primary Progressive Multiple Sclerosis	Rituximab	-		Not covered by the sponsor	13/1,045 (1.2 %)	
AG40852	Entrectinib	Solid tumours (NTRK)	No	-		Not covered by the sponsor	Not included in the analysis	
		Non-Small Cell Lung Cancer	Crizotinib	-		Used with the SoC	0/5	
AG40661	Polatuzumab Vedotin	Diffuse Large B-Cell Lymphoma	Bendamustine + rituximab (1) Lenalidomide (648 List) (2)	Bendamustine + rituximab	Covered by the sponsor	-	20/151 (13.2 %)	
AG41381	Risdiplam	Spinal Muscular Atrophy Type 1	Nusinersen	-		-	0/28	
AG42025	Risdiplam	Spinal Muscular Atrophy Type 2	No	-		-	Not included in the analysis	
AL41711	Trastuzumab emtansine	Breast Cancer	Trastuzumab + Capecitabina (1) Lapatinib + Capecitabina (2)	-		Not covered by the sponsor	0/621	
Total			2,713 patients with SoC	-		-	57/2,713 (2.1 %)	

* # of patients / % of patients recruited

Table 4 highlights the averted mean cost per patient due to avoided treatment with the SoC, and the mean incremental costs for hospitals due to the CUP program. The former ranges from €3,002 (rituximab for Primary Progressive Multiple Sclerosis) to €200,000 (nusinersen for Type-1 SMA). Diagnostic tests are annulled as either covered by the company or cancelled out since they would have also been used with the SoC. The three exceptions are represented by:

**Table 4:** Mean averted and incremental cost per patient due to each CUP

# of CUP	Molecule	Indication	Mean cost per patient of SoC				Sources	Mean cost per patient of combination drugs	Mean cost per patients of diagnostic test	Mean cost per (recruited) patient of side effects		Mean cost per (hospitalised) patient of side effects	
			**SoC1**	**SoC2**	**SoC3**	**SoC4**							
MO29499	Alectinib	Non-Small Cell Lung Cancer 1	€ 20,350	€ 44,860	-	-	SoC1 [42]; SoC2 [42, 43]	-	€ 106	-		-	
ML40066	Alectinib	Non-Small Cell Lung0 Cancer 2	€ 2,984	€ 9,700	€ 11,080	€ 14,500	SoC1 [44, 45] SoC2 and SoC3 [46] SoC4 [44]	-	-	-		-	
ML39740	Atezolizumab	Urothelial Carcinoma	€ 3,674	€ 12,902	€ 32,223	-	SoC1 [47, 48] SoC2 [49, 50] SoC^3^ [50, 73]	-	-	€ 60		€ 1,922	
AL41528	Atezolizumab	Non-Small Cell Lung Cancer	€ 9,395	€ 10,732	€ 33,229	-	SoC1 and SoC2 [46] SoC3 [51, 52, 72]	-	-	€ 11		€ 1,404	
AL41712	Atezolizumab	Triple-Negative Breast Cancer	€ 7,104	-	-	-	SoC1 [53, 54]	-	-	€ 90		€ 1,848	
M029476	Cobimetinib	Melanoma	€ 33,824	€ 39,991	€ 41,274	-	SoC1 [55] SoC2 [55, 56] SoC3 [55]	€ 16,086	-	€ 125		€ 2,029	
MA30130	Ocrelizumab	Primary Progressive Multiple Sclerosis	€ 3,002	-	-	-	SoC1 [57, 58]	-	€ 20	€ 27		€ 2,139	
AG40852	Entrectinib	Non-Small Cell Lung Cancer	€ 79,019	-	-	-	SoC1 [42]	-	-	-		-	
AG40661	Polatuzumab Vedotin	Diffuse Large B-Cell Lymphoma	€ 55,583	€ 56,658	-	-	SoC1 [59, 60, 69, 70, 71] SoC2 [60, 61, 62]	-	-	€ 151		€ 2,488	
AG41381	Risdiplam	Spinal Muscular Atrophy Type 1	€ 200,000	-	-	-	SoC1 [63]	-	-	-		-	
AL41711	Trastuzumab emtansine	Breast Cancer	€ 3,780	€ 19,576	-	-	SoC1 and SoC2 [64]	-	€ 253	-		-	
Mean costfor hospitalised patients													€ 2,184

- IHC (Immuno-Histo-Chemistry) and follow-up bilirubin and albumin protein for alectinib for adult patients with Anaplastic Lymphoma Kinase (ALK)-positive advanced Non-Small Cell Lung Cancer (NSCLC);
- Hepatitis B testing to identify patients at risk of reactivation with ocrelizumab;
- Fluorescent In-Situ Hybridization (FISH) for trastuzumab emtansine.

The mean cost per hospitalised patient due to adverse events amounts to €2,184, ranging from €1,404 to €2,488 (Table 4). As 57 out of 2,713 patients presented severe side effects resulting in hospitalisation, the mean cost of hospitalisation per patient recruited in CUP equals to €45.

The mean averted cost per patient due to avoided use of the SoC ranged from €11,415 to €20,299 and from €13,555 to €28,235 for all programs and cancer CUP, respectively. The mean incremental cost of all CUP (combination therapies, diagnostic tests, and hospitalisations due to side effects) amounted to €1,646, and to €2,694 for cancer CUP. Total savings for payers ranged from €26.5 million (€17.8 for cancer CUP) to €50.6 million (€41.9 million for cancer CUP) (Table 5). Mean savings per patient ranged from €10,861 to €25,559.

**Table 5:** Mean and total averted, incremental cost and net costs of CUP

*All programs (2,713 patients)*			
		**Min**	**Max**
**Averted cost**			
Mean cost per patient of SoC	a	€ 11,415	€ 20,299
Total costs of SoC	b	€ 30,967,593	€ 55,072,260
**Incremental cost**			
Mean cost per patient of CUP	c	€ 1,646 €	
Total cost of CUP	d	€ 4,466,187 €	
**Net cost**			
Net cost per patient	e=c-a	€ -9,769 €	€ -18,653
Total net costs	f=d-b	€ -26,501,406 €	€ -50,606,073
***Onco programs (1,640 patients)***			
		**Min**	**Max**
**Averted cost**			
Mean cost per patient of SoC	g	€ 13,555 €	€ 28,253
Total costs of SoC	h	€ 22,230,423 €	€ 46,335,090
**Incremental cost**			
Mean cost per patient of CUP	i	€ 2,694	
Total cost of CUP	j	€ 4,418,000	
**Net cost**			
Net cost per patient	k=i-g	€ -10,861	€ -25,559
Total net costs	l=j-h	€ -17,812,423	€ -41,917,090

Appendix 1 lists the averted costs, incremental costs, and net costs for each CUP. Net costs are mostly driven by the mean averted cost of the SoC, and the number of patients recruited in the CUP.

## Discussion

This paper has investigated, for the first time in the literature, the economic consequences of 11 CUP for medicines from the perspective of the NHS. Avoided costs of treating patients with the SoC, if existing and reimbursed, incremental costs of diagnostics, combination therapies, and side effects of CUT were included. Savings generated by the CUP ranged from €26.5 million (€9.8k per patient) to €50.6 million (€18.7k per patient), depending on the SoC used as an alternative to the CUT.

These findings cannot be fully compared with the economic impact of a CT. If Italian studies on CT avoided costs are considered, regarding only medicines [31, 32] and diagnostic procedures [30], the mean savings per patient treated are higher in our findings. This is mainly caused by the higher cost of the hypothetical SoC used in clinical practice.

It is important that policy makers and healthcare managers appropriately and carefully consider this evidence on the following grounds.

First, the findings represent costs avoided from the perspective of the NHS and not of society as a whole.

Secondly, incremental costs, despite quite negligible, are costs generated by CUP, whereas avoided costs are contingent upon the actual use of alternative treatments.

Thirdly, in this study costs of diagnostic testing and inpatient care have been estimated using the respective fee-for-service/episode, which represents a cost for the NHS, whereas, from the perspective of providers, it represents a revenue.

Based on the above, net savings from compassionate use should not justify delays in decision-making on pricing and reimbursement (P&R) upon approval of the medicine, on the grounds of shifting the burden from the payer to the industry. In principle, any early access program, like compassionate use, should terminate when the drug is approved. However, delays in P&R decisions [65] have resulted in pharmaceutical companies prolonging CUP, to avoid interruptions in the continuity of care of patients. Further, companies should not expect to secure reimbursement and/or a higher price for their medicines on the grounds of the latter’s sponsorship of a CUP. As not all medicines used in CUP are necessarily approved for reimbursement in Italy, prices should reflect value [66] and not whether the relevant medicine has been granted early access through CUP.

Our study has some limitations

The first and most important limitation is that we did not estimate the net economic impact of the CUT on disease progression compared to the SoC. This was because CUP are not accompanied by systematic collection of data on effectiveness and resource consumption (apart from diagnostic testing and hospitalisations due to adverse events). Furthermore, had this data been available, they would have required extrapolation beyond the duration of CUP. This extrapolation, besides being out of scope, would have implied a case-control simulation, where for the ‘control’ arm (SoC or no treatment) data were not available. Another option would have been to adapt published cost-effectiveness analyses on patients treated in CUP through micro-simulation. However the evidence from cost-effectiveness studies is limited, for Italy, only to one study [67].

Secondly, our analysis adopted the perspective of the NHS. To this end, the impact of CUP on other third payers (e.g., social insurance schemes for missed work) and patients and their relatives (e.g., remunerated / informal care provided) was not considered. Indeed, for some diseases, like multiple sclerosis, costs beyond healthcare may play an important role.

Thirdly, since the comparative impact on disease progression and future cost avoidance was not included, we did not conduct an economic impact analysis where no alternative to the CUT was available. The scientific understanding of key molecular pathways and the development of new molecular entities in most of the diseases targeted by the examined CUP present opportunities to fill unmet needs, even in areas with existing alternatives.

In addition, the absence of long-term follow-up of patients recruited in CUP did not allow for the evaluation of the CUP effects on therapeutic sequencing. It may happen that the SoC is used after the CUP, or vice versa when patients are not recruited in the CUP (i.e., they are first treated with the SoC and afterwards with the CUT, once it has been approved for reimbursement).

Furthermore, our analysis did not include an estimate of severe adverse events costs for the SoC, normally retrieved from the literature (unless it was used as a comparator in trials for the CUT). This implies an overestimation of the net economic burden of side effects of CUP.

We were also not always able to include the effect of discounts and MEA in the calculation of the cost of the SoC.

The costs of nusinersen for Type-1 Spinal Muscular Atrophy refer to data from the first year of treatment, since real world data on treatment duration beyond the first year were not available.

Incremental costs for diagnostic procedures were estimated on the grounds of the presumed clinical practice with the SoC and assuming maximum impact for the NHS. For example, we decided to include (i) IHC to detect ALK-positive patients, despite its use with crizotinib as an alternative to alectinib for adult patients with advanced NSCLC and (ii) FISH for trastuzumab emtansine, despite its use with trastuzumab as a neo-adjuvant therapy. If the cost of these two diagnostic procedures were eliminated, the calculated incremental cost would decrease from €4.5 million to €4.3 million with a net economic benefit ranging from €26.7 million (instead of €26.5) to €50.8 million (instead of €50.6 million).

Finally, while different SoC were identified, we did not have any information on their use in clinical practice. This information could have allowed for the calculation of a weighted mean cost per patient receiving the respective SoC. Thus, we were only able to provide a minimum and maximum average averted cost per patient, where the actual mean cost depends on the present market share of each single SoC.

Despite these limitations, this paper constitutes the first evidence on the economic advantages of medicines provided for compassionate use.

## Conclusions

CUP have been introduced to guarantee earlier access to medicines for patients not recruited in trials, responding to the ethical imperative of addressing unmet needs. In this paper, we have demonstrated that CUP have important advantages also from an economic standpoint. Beyond their economic impact, they can serve as an important source of data, enhancing information availability during the approval and P&R processes. This, however, would imply more systematic data collection on clinical outcomes and on the impact from the perspective of patients, as well as on health resources. Such data collection would also inform a more comprehensive economic evaluation of these programs.

In conclusion, CUP for medicines represent an opportunity to accelerate patient access to medicines for rare and severe diseases, in cases where patients have not been recruited or are not eligible for recruitment in clinical trials. For clinicians who have not been involved in clinical trials, they represent an opportunity to familiarise with medicines likely to become available in clinical practice. For health care payers they present cost saving opportunities, while the impact and savings could be larger if the societal perspective is considered.

## Supplementary information

**Additional file 1**

## Data Availability

The datasets used and/or analysed during the current study are available from the corresponding author on reasonable request.

## Abbreviations

ALK: Anaplastic Lymphoma Kinase; CT: Clinical Trial(s)
CUP: Compassionate Use Program(s)
CUT: Compassionate Use Treatment(s)
DCA: Drug Cost Avoidance
FISH: Fluorescent In Situ Hybridization
DLBCL: Diffuse Large B-Cell Lymphoma
DRG: Diagnosis-Related Group
IHC: Immuno-Histo-Chemistry
MEA: Managed Entry Agreements
MedDRA: Medical Dictionary for Regulatory Activities
NSCLC: Non-Small Cell Lung Cancer
NTRK: Neurotrophic Tyrosine Receptor Kinase
PCA: Pathology Cost Avoidance
P&R: Pricing & Reimbursement
PPMS: Primary Progressive Multiple Sclerosis
SMA: Spinal Muscular Atrophy
SmpC: Summary of Product Characteristics
SoC: Standard of Care
TNBC: Triple-Negative Breast Cancer
UC: Urothelial Carcinoma
