# Supplementary material for: The economic impact of compassionate use of medicines
Source: BMC Health Serv Res. 2021 Dec 4;21:1303. doi: 10.1186/s12913-021-07255-w (PMC8645125; doi:10.1186/s12913-021-07255-w)
Supplement: Supplementary file 1 — Additional file 1 [file 12913_2021_7255_MOESM1_ESM.docx]

Appendix 1: Mean and total averted, incremental cost and net costs of each CUP program

| **MO29499 - Alectinib Non-Small Cell Lung Cancer 1** |  | **Min** | **Max** |
| --- | --- | --- | --- |
| Mean cost per patient of SoC | a | € 20,350 | € 44,860 |
| Total costs of SoC | b | € 427,350 | € 942,060 |
| Mean cost per patient of CUP | c | € 106 | |
| Total cost of CUP | d | € 2,232 | |
| Net cost per patient | e=c-a | € -20,244 | € -44,754 |
| Total net costs | f=d-b | € -425,118 | € -939,828 |
| **ML39740 - Atezolizumab Urothelial Carcinoma** |  |  |  |
| Mean cost per patient of SoC | a | € 3,674 | € 32,223 |
| Total costs of SoC | b | € 815,602 | € 7,153,506 |
| Mean cost per patient of CUP | c | € 60 | |
| Total cost of CUP | d | € 13,430 | |
| Net cost per patient | e=c-a | € -3613 | € -32,163 |
| Total net costs | f=d-b | € -80,2172 | € -7,140,076 |
| **AL41712 - Atezolizumab Triple-Negative Breast Cancer** | |  |  |
| Mean cost per patient of SoC | a | € 7,104 | |
| Total costs of SoC | b | € 291,279 | |
| Mean cost per patient of CUP | c | € 0 | |
| Total cost of CUP | d | € 0 | |
| Net cost per patient | e=c-a | € -7,104 | |
| Total net costs | f=d-b | € -291,279 | |
| **MA30130 - Ocrelizumab Primary Progressive Multiple Sclerosis** | |  |  |
| Mean cost per patient of SoC | a | € 3,002 | |
| Total costs of SoC | b | € 3,137,169 | |
| Mean cost per patient of CUP | c | € 46 | |
| Total cost of CUP | d | € 48,188 | |
| Net cost per patient | e=c-a | € -2,956 | |
| Total net costs | f=d-b | € -3,088,982 | |
| **AG40661 - Polatuzumab Diffuse Large B-Cell Lymphoma** | |  |  |
| Mean cost per patient of SoC | a | € 55,583 | € 56,658 |
| Total costs of SoC | b | € 8,393,041 | € 8,555,298 |
| Mean cost per patient of CUP | c | € 330 | |
| Total cost of CUP | d | € 49,757 | |
| Net cost per patient | e=c-a | € -55,254 | € -56,328 |
| Total net costs | f=d-b | € -8,343,284 | € -8,505,541 |
| **AL41711 - Trastuzumab Emt. Breast Cancer** |  |  |  |
| Mean cost per patient of SoC | a | € 3,780 | € 19,576 |
| Total costs of SoC | b | € 2,347,380 | € 12,156,761 |
| Mean cost per patient of CUP | c | € 253 | |
| Total cost of CUP | d | € 157,250 | |
| Net cost per patient | e=c-a | € -3,527 | € -19,323 |
| Total net costs | f=d-b | € -2,190,130 | € -11,999,511 |
| **MO40066 - Alectinib Non-Small Cell Lung Cancer 2** |  |  |  |
| Mean cost per patient of SoC | a | € 2,984 | € 14,500 |
| Total costs of SoC | b | € 674,409 | € 3,277,000 |
| Mean cost per patient of CUP | c | € 0 | |
| Total cost of CUP | d | € 0 | |
| Net cost per patient | e=c-a | € -2,984 | € -14,500 |
| Total net costs | f=d-b | € -674,409 | € -3,277,000 |
| **AL41528 - Atezolizumab Non-Small Cell Lung Cancer** | |  |  |
| Mean cost per patient of SoC | a | € 9,395 | € 33,229 |
| Total costs of SoC | b | € 1,174,393 | € 4,153,618 |
| Mean cost per patient of CUP | c | € 3,994 | |
| Total cost of CUP | d | € 499,247 | |
| Net cost per patient | e=c-a | € -5,401 | € -29,235 |
| Total net costs | f=d-b | € -675,146 | € -3,654,371 |
| **M029746 - Cobimetinib Melanoma** |  |  |  |
| Mean cost per patient of SoC | a | € 33,824 | € 41,274 |
| Total costs of SoC | b | € 7,711,872 | € 9,410,472 |
| Mean cost per patient of CUP | c | € 16,211 | |
| Total cost of CUP | d | € 3,696,084 | |
| Net cost per patient | e=c-a | € -17,613 | € -25,063 |
| Total net costs | f=d-b | € -4,015,788 | € -4,015,788 |
| **AG40852 - Entrectinib (Non-Small Cell Lung Cancer)** | |  |  |
| Mean cost per patient of SoC | a | € 79,019 | |
| Total costs of SoC | b | € 395,097 | |
| Mean cost per patient of CUP | c | € 0 | |
| Total cost of CUP | d | € 0 | |
| Net cost per patient | e=c-a | € -79,019 | |
| Total net costs | f=d-b | € -395,097 | |
| **AG41381 - Risdiplam (Spinal Muscular Atrophy Type 1** | |  |  |
| Mean cost per patient of SoC | a | € 200,000 | |
| Total costs of SoC | b | € 5,600,000 | |
| Mean cost per patient of CUP | c | € 0 | |
| Total cost of CUP | d | € 0 | |
| Net cost per patient | e=c-a | € -200,000 | |
| Total net costs | f=d-b | € -5,600,000 | |
